# Longitudinal loop diuretic use in patients with HFrEF initiated with sodium–glucose cotransporter 2 inhibitors

**DOI:** 10.1093/ehjcvp/pvag041

**Published:** 2026-06-10

**Authors:** Daniela Tomasoni, Lina Benson, Javed Butler, Federica Guidetti, Michael Melin, Carin Corovic Cabrera, Marco Metra, Lars H Lund, Gianluigi Savarese

**Affiliations:** Department of Clinical Science and Education, Södersjukhuset, Karolinska Institutet, Sjukhusbacken 10, Stockholm 118 83, Sweden; Cardiology–ASST Spedali Civili di Brescia and Department of Medical and Surgical Specialties, Radiological Sciences, and Public Health, University of Brescia, 25123 Brescia, Italy; Department of Clinical Science and Education, Södersjukhuset, Karolinska Institutet, Sjukhusbacken 10, Stockholm 118 83, Sweden; Department of Medicine, Division of Cardiology, Karolinska Institutet, SE-171 76 Stockholm, Sweden; Baylor Scott and White Research Institute, 3434 Live Oak St Ste 501, Dallas, TX 75204, USA; Department of Medicine, University of Mississippi 2500 N State St, Jackson, MS 39216, USA; Department of Clinical Science and Education, Södersjukhuset, Karolinska Institutet, Sjukhusbacken 10, Stockholm 118 83, Sweden; Department of Cardiology, University Cardiovascular Centre, Bern University Hospital, Inselspital, 3010 Bern, Switzerland; Department of Clinical Science and Education, Södersjukhuset, Karolinska Institutet, Sjukhusbacken 10, Stockholm 118 83, Sweden; Department of Clinical Science and Education, Södersjukhuset, Karolinska Institutet, Sjukhusbacken 10, Stockholm 118 83, Sweden; Cardiology–ASST Spedali Civili di Brescia and Department of Medical and Surgical Specialties, Radiological Sciences, and Public Health, University of Brescia, 25123 Brescia, Italy; Department of Medicine, Division of Cardiology, Karolinska Institutet, SE-171 76 Stockholm, Sweden; Department of Clinical Science and Education, Södersjukhuset, Karolinska Institutet, Sjukhusbacken 10, Stockholm 118 83, Sweden

**Keywords:** HFrEF, SGLT2 inhibitors, Loop diuretics, Furosemide, Use, Dose

## Abstract

**Aims:**

A reduction in loop diuretics need after sodium–glucose cotransporter-2 inhibitors (SGLT2i) initiation has been suggested by retrospective analyses of clinical trials. We aimed to investigate long-term changes in loop diuretic therapy after SGLT2i initiation in an unselected, real-world heart failure (HF) cohort.

**Methods and results:**

Patients with HF with reduced ejection fraction (HFrEF) enrolled in the SwedeHF between January 2020 and October 2021 were considered. Patients initiated with SGLT2i were the cases. Controls did not initiate SGLT2i despite having an indication. Trajectories in daily furosemide-equivalent doses from baseline to 1-year follow-up were compared in cases vs. controls. Overall, 5623 (81.6%) controls and 1265 (18.4%) cases were analysed. At baseline, 5224 (75.8%) patients were prescribed with loop diuretics with a mean (SD) furosemide-equivalent dose of 36.7 (27.4) mg. SGLT2i users, compared with controls, more likely experienced a loop diuretic withdrawal/dose reduction (difference between proportions 6% [3%; 9%] *P*-value <0.001), and less likely had a loop diuretic initiation or dose increase (difference between proportions −5% [−7%; −3%], *P*-value <0.001), leading to a more accentuated net decrease in furosemide-equivalent daily doses (adjusted mean difference between groups −4.2 [−7; −1.5] mg, *P*-value 0.002). In a multivariable regression model, the adjusted odds ratio (OR) for decrease in loop diuretic doses/withdrawn after SGLT2i initiation was 1.58 (1.35–1.85), and the adjusted OR for increase in loop diuretic doses/new initiation was 0.50 (0.38–0.64).

**Conclusion:**

In this real-world population with HFrEF, SGLT2i initiation was associated with a decrease in loop diuretic need at 1-year follow-up.

## Graphical Abstract

**Graphical Abstract:**
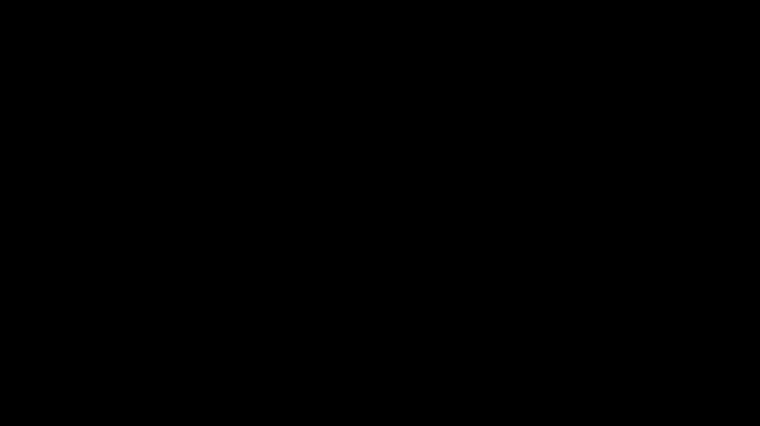
Study design and main results of the study. Among patients with HFrEF from the SwedeHF, SGLT2i initiation was associated with a decrease in loop diuretic need at 1-year follow-up. CI, confidence interval; eGFR, estimated glomerular filtration rate; HFrEF, heart failure with reduced ejection fraction; OR, odds ratio; SGLT2i, sodium–glucose cotransporter-2 inhibitors; T1DM, type 1 diabetes mellitus.

## Introduction

Sodium–glucose cotransporter-2 inhibitors (SGLT2i) reduced the risk of cardiovascular death or heart failure (HF) events, and led to an improvement in health-related quality of life in patients with HF across the entire spectrum of ejection fraction (EF).^1,2^

SGLT2 inhibition prevents the absorption of sodium and glucose in the proximal renal tubule, resulting in enhanced natriuresis and glucosuria, accompanying water excretion. Hence, among others, one possible hypothesis explaining the early impact of SGLT2i on clinical outcomes observed within few weeks after initiation in randomized controlled trials (RCTs) is their diuretic effect,^3,4^ leading to effective and sustained decongestion. On the other hand, a large diuretic or natriuretic effect with SGLT2i has not generally been observed in the longer-term, which might be explained by a rapid renal adaptation to these drugs, due to counter-regulatory mechanisms, that occurs within a few days or weeks, preventing progressive volume losses.^5,6^

However, the addition of an SGLT2i might lead to a reduction in required daily diuretic doses or even withdrawal of diuretic therapy through several mechanisms, including the preservation of renal and cardiac function.^1,4^ Secondary analyses of RCTs have suggested that administration of SGLT2i may be associated with less likelihood of loop diuretics initiation or dose increase and a greater likelihood of their discontinuation.^7-10^ These data were shown in patients selected to participate in RCTs. Evidence for an unselected real-world cohort is lacking.

Thus, the objective of the present study was to assess real-world long-term changes in diuretic requirement after the initiation of SGLT2i, by comparing patterns in loop diuretics use at baseline vs. 1 year after SGLT2i initiation with those from a control cohort with an indication but not initiated with SGLT2i, in the nationwide population with HF and reduced EF (HFrEF) from the Swedish HF Registry (SwedeHF).

## Methods

### Data sources

The study population was derived from the SwedeHF, which has been previously described.^11^ Briefly, SwedeHF is an ongoing nationwide health quality and research registry that includes in- and out-hospital patients with HF. Coverage of SwedeHF in 2021 was ∼30% of the prevalent HF population in Sweden.^12^ The registry includes HF patients regardless of EF, with EF collected in most patients as a categorical variable (i.e. < 40%, 40%–49%, >50%). Therefore, we could define HFrEF as EF < 40% and not as ≤40% according to the Universal Definition of HF.

For this study, SwedeHF was linked with (i) the National Prescribed Drug Register, providing information on the prescribed drugs and related doses as dispensed in pharmacies; (ii) the National Patient Register, providing additional data on comorbidities; (iii) the Cause of Death Registry, providing the date and cause of death; (iv) the longitudinal integrated database for health insurance and labour market studies and the Register of the Total Population, providing data on the socioeconomic factors.

Linkage between these registries was allowed by the personal identification number, which all residents in Sweden have. Data source and definition for each variable was reported in Supplementary material online, *Table S1*.

This analysis including the linkage across several registries was approved by the Swedish Ethical Review Authority and complies with the Declaration of Helsinki. Individual patient consent was not required, but patients are informed of entry into SwedeHF and can opt out.

### Study population

A flowchart summarizing the cohort selection process is reported in Supplementary material online, *Figure S1*. We selected patients with HFrEF, without type-1 diabetes mellitus, and with estimated glomerular filtration rate (eGFR) ≥ 20 mL/min/1.73 m^2^ (i.e. with indication for SGLT2i in HFrEF). As in Sweden SGLT2i were firstly approved for the treatment of HFrEF in 2020, the timing of inclusion was restricted from 2020 onwards. We assessed changes in loop diuretic use at 12 (+/−) 2 months (see Supplementary material online, *Figure S2*). To ensure that all included patients had a 4-month time window at follow-up, we excluded those who died before 14 months of follow-up or had <14 months between their first registration and the end of follow-up. Follow-up data were available until 31 December 2022, resulting into a final cohort included in SwedeHF between 1 January 2020 and 31 October 2021.

Patients on prevalent use of SGLT2i at the index date, i.e. > 2 months prior to the index date, or those on SGLT2i other than empagliflozin/dapagliflozin, as they are the only SGLT2i to have guideline recommendation for HFrEF, were excluded.

Patients in the SwedeHF registry can be registered more than once. If the same patient was registered more than once, we selected the first record after applying the inclusion criteria.

### Definitions

#### Baseline

Index date was defined as the date of registration in SwedeHF, i.e. the date of the outpatient visit for outpatients or the date of discharge for inpatients. Initiation of SGLT2i and use of loop diuretics at baseline were evaluated within 2 months before/after the index date in SwedeHF through dispended prescriptions in the National Prescribed Drug Register (see Supplementary material online, *Figure S2*). If a patient had multiple dispensed prescriptions within the 4-month time window, the dispensation closest to baseline was selected.

#### Follow-up

Use of SGLT2i and loop diuretics at follow-up was evaluated at 12 +/− 2 months through the dispensed prescriptions in National Prescribed Drug Registry (see Supplementary material online, *Figure S2*). If a patient had multiple dispensed prescriptions within the 4-month time window, the dispensation closest to 12 months was selected.

#### Loop diuretics

We used furosemide-equivalent daily doses for loop diuretics. Bumetanide 1 mg and torsemide 20 mg were considered equivalent to furosemide 80 mg orally as in previous studies.^7,8^

Additional information about the dose calculation is reported in Supplementary material online, *Table S1*.

#### Cases and controls

Patients initiated with a recommended SGLT2i (dapagliflozin or empagliflozin) at baseline (index date +/− 2 months) were considered as cases (i.e. SGLT2i new users). The control population consisted of patients with indication but not initiated with SGLT2i at baseline during the same time period.

### Statistical analysis

Baseline characteristics of the study population are presented for SGLT2i new users (cases) vs. controls. Patient characteristics were reported as median [interquartile range (IQR)] if continuous, and as counts (percentages) if categorical, and compared by Kruskal–Wallis and χ^2^ tests, respectively.

Mean (SD) of loop diuretic doses at baseline and at follow-up were reported and compared between cases vs. controls using *t*-test. Daily loop diuretic dose for patients not prescribed with a loop diuretic was considered 0 mg. The delta dose of loop diuretics (dose at follow-up—dose at baseline) was calculated and reported as mean (SD). As mean doses of loop diuretics at baseline were slightly different between cases and controls, we also reported the percentage change in doses using as a denominator the baseline dose (percentage change calculated as delta dose/baseline dose). An additional analysis assessing changes in loop diuretic doses stratified by categorized baseline furosemide doses (i.e. <40 mg, 40–79 mg, ≥80 mg) was performed.

A multivariable linear regression analysis was performed to examine the relationship between SGLT2i initiation and change in loop diuretic doses over time. The model included SGLT2i initiation, baseline furosemide-equivalent doses and all variables marked with ^a^ in *Table 1* as independent variables and the delta dose of loop diuretics between baseline to follow-up as the dependent variable. The estimated mean difference [95% confidence interval (CI)] in loop diuretic dose changes among SGLT2i new users vs. controls was derived. An exploratory analysis was performed to assess potential effect modification by SGLT2i type (dapagliflozin vs. empagliflozin). The change in use/dose of loop diuretics between baseline and 1-year follow-up was then categorized and defined as: (i) stable: if the patient was/was not on treatment at baseline and at follow-up, or was stable in daily doses; (ii) increased: if the patient was not on loop diuretics at baseline and was on loop diuretics at follow-up, or increased in dose; (iii) decreased: if the patient was on loop diuretics at baseline but was not at follow-up, or decreased in dose. For facilitating statistical modelling, dichotomous variables were created: ‘Decrease’ vs. ‘No Decrease’ (i.e. Decrease vs. Stable/Increase) and ‘Increase’ vs. ‘No Increase’ (i.e. Increase vs. Stable/Decrease).

**Table 1:** Baseline characteristics stratified by SGLT2 inhibitors initiation

Variable	No SGLT2i	SGLT2i new users	*P*-value
*n*	5623 (81.6%)	1265 (18.4%)	
Sex (male)^a^	4007 (71.3%)	967 (76.4%)	<0.001
Age^a^ (≥80 years)	1393 (24.8%)	196 (15.5%)	<0.001
Calendar year			
2020	3177 (56.5%)	235 (18.6%)	<0.001
2021	2446 (43.5%)	1030 (81.4%)	
Inpatient^a^	840 (14.9%)	192 (15.2%)	0.83
HF hospitalization (within 12 months)^a^	2534 (45.1%)	617 (48.8%)	0.017
Duration of HF (≥6 months)^a^	2439 (44.2%)	511 (40.8%)	0.031
NYHA class^a^			
I–II	3049 (63.8%)	653 (60.2%)	0.029
III–IV	1731 (36.2%)	431 (39.8%)	
BMI (≥30 kg/m^2^)^a^	1190 (25.7%)	335 (32.7%)	<0.001
SBP (≥120 mmHg)^a^	3127 (57.8%)	644 (52.8%)	0.001
Heart rate (>70 bpm)^a^	2846 (52.9%)	654 (54.0%)	0.47
eGFR (<60 mL/min/1.73 m2)^a^	1728 (31.6%)	336 (26.9%)	0.001
Potassium^a^			
Normakalemia	5129 (94.2%)	1160 (93.3%)	0.36
Hypokalemia	129 (2.4%)	38 (3.1%)	
Hyperkalemia	189 (3.5%)	45 (3.6%)	
Haemoglobin (≥13.7 g/dL)^a^	2544 (51.8%)	628 (55.2%)	0.037
NT-proBNP (ng/L)	2111.5 (923.0, 4621.0)	1710.0 (848.5, 3822.0)	<0.001
NT-proBNP (≥2000 ng/L)^a^	2471 (51.9%)	497 (44.1%)	<0.001
RASi/ARNI^a^	5348 (95.2%)	1229 (97.2%)	0.002
Beta-blockers^a^	5269 (93.8%)	1206 (95.3%)	0.033
MRA^a^	3020 (53.8%)	878 (69.4%)	<0.001
Digoxin^a^	488 (8.7%)	99 (7.8%)	0.33
Antiplatelet^a^	1719 (30.6%)	406 (32.1%)	0.30
Anticoagulant^a^	3062 (54.5%)	661 (52.3%)	0.14
Statin^a^	2936 (52.3%)	734 (58.1%)	<0.001
Nitrate^a^	345 (6.1%)	54 (4.3%)	0.010
CRT/ICD^a^	827 (14.7%)	217 (17.2%)	0.029
Hypertension^a^	3588 (63.8%)	806 (63.7%)	0.95
Diabetes^a^	1123 (20.0%)	471 (37.2%)	<0.001
Ischaemic heart disease^a^	2667 (47.4%)	618 (48.9%)	0.36
Atrial fibrillation^a^	3002 (53.4%)	599 (47.4%)	<0.001
Valvular heart disease^a^	1164 (20.7%)	263 (20.8%)	0.94
COPD^a^	589 (10.5%)	110 (8.7%)	0.058
Liver disease^a^	133 (2.4%)	34 (2.7%)	0.50
PAD^a^	453 (8.1%)	94 (7.4%)	0.46
Previous stroke/TIA^a^	745 (13.2%)	153 (12.1%)	0.27
History of cancer^a^	623 (11.1%)	115 (9.1%)	0.039
Family type^a^			
Cohabitating	3112 (55.4%)	702 (55.6%)	0.91
Living alone	2505 (44.6%)	561 (44.4%)	
Follow-up location^a^			
Primary care/other	697 (12.6%)	57 (4.6%)	<0.001
Hospital	4835 (87.4%)	1178 (95.4%)	
Follow-up HF unit	5094 (93.2%)	1188 (97.2%)	<0.001

ARNI, angiotensin receptor–neprilysin inhibitor; BMI, body mass index; COPD, chronic obstructive pulmonary disease; CRT, cardiac resynchronization therapy; eGFR, estimated glomerular filtration rate; HF, heart failure; ICD, implantable cardiac defibrillator; MRA, mineralocorticoid receptor antagonist; NYHA, New York Heart Association; NT-proBNP, N-terminal pro-B-type natriuretic peptide; PAD, peripheral artery disease; RASi, renin–angiotensin system inhibitors; SBP, systolic blood pressure; SGLT2i, sodium–glucose cotransporter-2 inhibitors; TIA, transient ischaemic attack.

^a^Variables that were included in the multivariable Cox regression model and logistic regression analyses.

The proportions of increase, decrease, or stable use of loop diuretics were compared in cases vs. controls by χ^2^ test. Logistic regression analysis was used to assess the association between SGLT2i initiation and the outcome of interest (decrease or increase in loop diuretic dosing/use). Logistic regression models were fitted as follows: (i) unadjusted and (ii) adjusted for variables marked with ^a^ in *Table 1* together with the baseline furosemide-equivalent dose. Odds ratio (ORs) and 95% CI were calculated.

As cases could discontinue SGLT2i during follow-up and controls could initiate SGLT2i during follow-up, we performed both an intention-to-treat analysis, i.e. SGLT2i status defined as at the index date, and a per-protocol analysis where we excluded cases discontinuing SGLT2i during the follow-up and controls who started a SGLT2i > 60 days after the index registration.

Missing data in multivariable models were handled by multiple imputation by chained equations (with 10 imputation), with Rubin’s rules used for combining estimates and standard errors across the imputed datasets. The imputation model included all variables used in the multivariable analyses, including the outcome variable, exposure, and relevant baseline covariates (as indicated with ^a^ in *Table 1*). Frequencies of missing data for each variable were reported in Supplementary material online, *Table S1*. The overall proportion of missing data was relatively low (and generally < 10%). Therefore, 10 imputations were considered adequate.

All analyses were performed using STATA version 16.1. The level of significance was set to 5%, two-sided. The study is reported in accordance with the RECORD-PE guidelines.

## Results

A flowchart reporting patient selection is reported in Supplementary material online, *Figure S1*. After applying the study selection criteria 6888 patients with HFrEF, of whom 1265 (18.4%) were cases and 5623 (81.6%) were controls, were analyzed. Median age was 72 (IQR 63–79) years and 4974 (72.2%) were males. Among SGLT2i new users, 915 (72%) and 350 (28%) initiated dapagliflozin and empagliflozin, respectively.

### Baseline characteristics (*Table 1*)

SGLT2i new users were younger, more likely males, with worse symptoms (i.e. higher NYHA functional class), lower systolic blood pressure but higher eGFR. They were also more likely to have a history of a HF hospitalization within 12 months. Obesity and type 2 diabetes were more prevalent among SGLT2i new users. Renin–angiotensin system inhibitors or angiotensin receptor neprilysin inhibitor, beta-blockers, and mineralocorticoid receptor antagonist were more likely prescribed in SGLT2i new users.

### Changes in furosemide-equivalent daily doses from baseline to 1-year follow-up

Overall, 5224 patients (75.8% of the total population) were prescribed with loop diuretics (any dose) at baseline, and mean (SD) furosemide-equivalent dose was 36.7 (27.4) mg.

At baseline, 67.4% cases vs. 77.7% controls were on loop diuretics, whereas 32.3% vs. 52.3% were on loop diuretics at 1-year follow-up (both *P*-value <0.001) (*Table 2*). SGLT2i new users were prescribed with lower furosemide-equivalent doses at baseline and at follow-up. Changes in mean doses (follow-up—baseline) were larger in SGLT2i new users vs. controls (−16.5 [30.3] vs. −11.9 [32.0] mg, between-group difference −4.6 [−6.5; −2.7] mg; *P*-value <0.001) (*Table 2*).

**Table 2:** Detailed description of loop-diuretic prescription

Variable	All	No SGLT2i	SGLT2i new users	Between-group difference	*P*-value
*n*	6888 (100%)	5623 (81.6%)	1265 (18.4%)		
On loop diuretic at baseline	5224 (75.8%)	4371 (77.7%)	853 (67.4%)	−10.3% (−13.1%; −7.5%)	<0.001
Furosemide-equivalent dose baseline (mg), mean (SD)	36.7 (27.4)	37.6 (27.0)	32.8 (28.5)	−4.9 (−6.5; −3.2)	<0.001
On loop diuretic at follow-up	3351 (48.6%)	2943 (52.3%)	408 (32.3%)	−20.1% (−23.0%; −17.2%)	<0.001
Furosemide-equivalent dose follow-up (mg), mean (SD)	24.0 (28.8)	25.7 (28.9)	16.3 (27.4)	−9.5 (−11.2;−7.7)	<0.001
Difference dose (follow-up—baseline) (mg), mean (SD)	−12.8 (31.2)	−11.9 (32.0)	−16.5 (30.3)	−4.6 (−6.5; −2.7)	<0.001
Difference dose in percentage (%), mean (SD)	−30 (60)	−30 (60)	−40 (60)	−8.8 (−12.2; −5.4)	<0.001
Difference loop diuretic dosing (follow-up—baseline), categories					<0.001
No changes	3403 (49.4%)	2790 (49.6%)	613 (48.5%)	−1.2% (−4.2%; 1.9%)	
Reduced/withdrawn	2669 (38.7%)	2114 (37.6%)	555 (43.9%)	6.3% (3.2%; 9.3%)	
Increased/new initiation	816 (11.8%)	719 (12.8%)	97 (7.7%)	−5.1% (−6.8%; −3.4%)	

SD, standard deviation; SGLT2i, sodium–glucose co-transporter 2 inhibitors.

**Figure 1:**
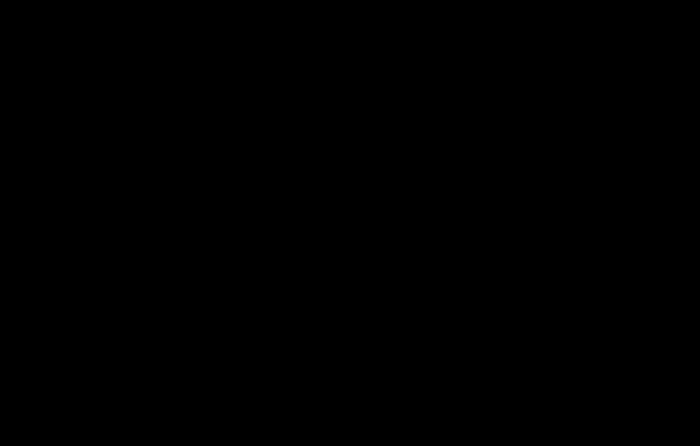
Changes in loop diuretic doses from baseline to 1-year follow-up. Error-bar represents 95% confidence interval. Linear regression analysis showed SGLT2i new users experienced a larger decrease in daily loop diuretic doses. SGLT2i, sodium–glucose co-transporter 2 inhibitors.

At multivariable linear regression analysis, SGLT2i new users experienced a larger decrease in daily loop diuretic doses between baseline and 1-year follow-up as compared with those not initiated with SGTL2i (adjusted estimated mean difference −4.2 [−7.0; −1.5] mg, *P*-value 0.002) (*Figure 1*). No significant interaction was observed between SGLT2i type (dapagliflozin vs. empagliflozin) and changes in loop diuretic doses (*P* for interaction = 0.7397). The results were confirmed when using percentage delta doses rather than absolute change in doses as dependent variable.

Patients initiated with SGLT2i, as compared with controls, more likely experienced a loop diuretic discontinuation or dose reduction (43.9% vs. 37.6%, difference between proportions 6.3% [3.2%; 9.3%], *P*-value <0.001), and less likely had a loop diuretic initiation or dose increase (7.7% vs. 12.8%, difference between proportions −5.1% [−6.8%; −3.4%], *P*-value <0.001) (*Table 2*).

**Figure 2:**
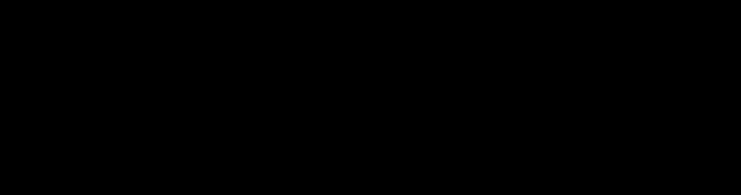
Changes in loop diuretic doses from baseline to follow-up stratified by baseline dose categories (i.e. <40 mg, 40–79 mg, ≥80 mg). SGLT2i, sodium–glucose co-transporter 2 inhibitors.

*Figure 2* illustrates distinct patterns across baseline loop diuretic dose categories. Patients receiving low baseline doses of loop diuretics (i.e. <40 mg) appeared less likely to increase their dose following SGLT2i initiation, whereas those on intermediate doses (40–79 mg) showed the greatest reductions. In contrast, patients on high baseline doses (≥80 mg) exhibited smaller between-group differences. Consistent results were also observed, after dichotomization, at the univariable logistic regression analysis with an OR 1.30 (95% CI 1.15–1.47) for decrease dose/loop diuretic withdrawn vs. stable/increase dose, and OR 0.57 (95% CI 0.45–0.71) for increase dose/new loop diuretic initiation vs. stable/decrease dose.

**Figure 3:**
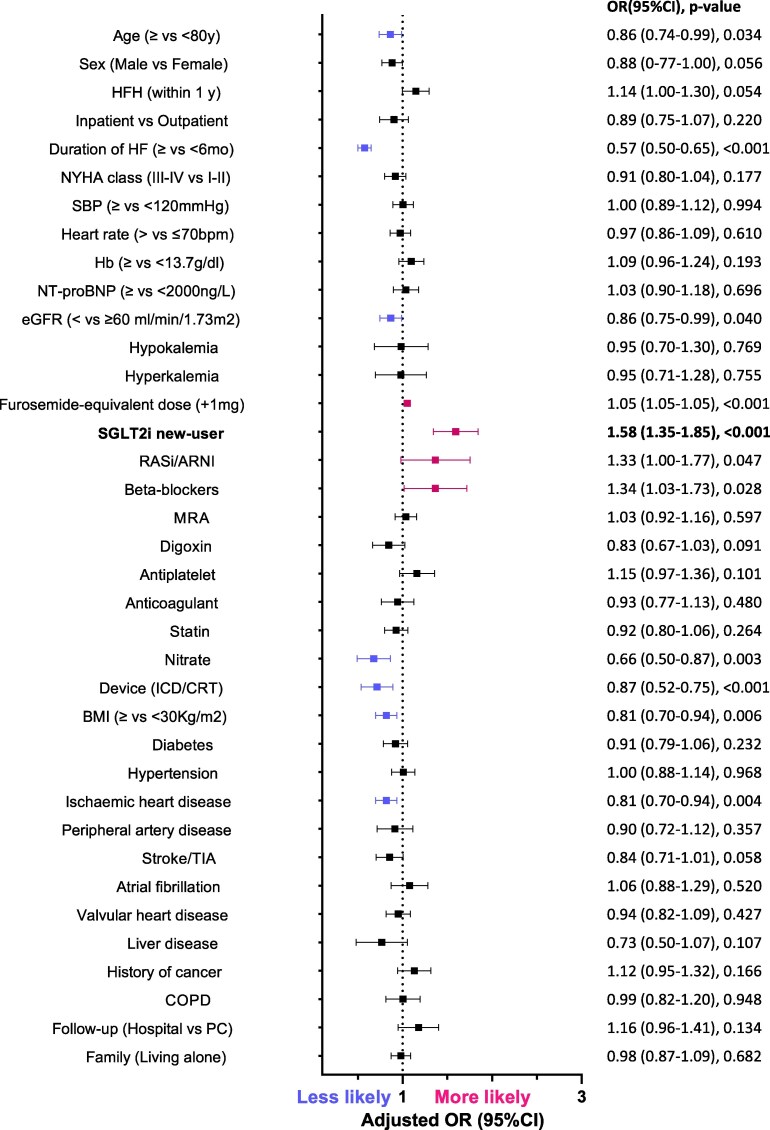
Predictors of decrease in loop diuretic use/dosing at follow-up. ARNI, angiotensin receptor–neprilysin inhibitor; BMI, body mass index; CI, confidence interval; COPD, chronic obstructive pulmonary disease; CRT, cardiac resynchronization therapy; eGFR, estimated glomerular filtration rate; Hb, haemoglobin; HF, heart failure; HFH, heart failure hospitalization; ICD, implantable cardiac defibrillator; MRA, mineralocorticoid receptor antagonist; NYHA, New York Heart Association; NT-proBNP, N-terminal pro-B-type natriuretic peptide; OR, odds ratio; PC, primary care; RASi, renin–angiotensin system inhibitors; SBP, systolic blood pressure; SGLT2i, sodium–glucose co-transporter 2 inhibitors; TIA, transient ischaemic attack.

**Figure 4:**
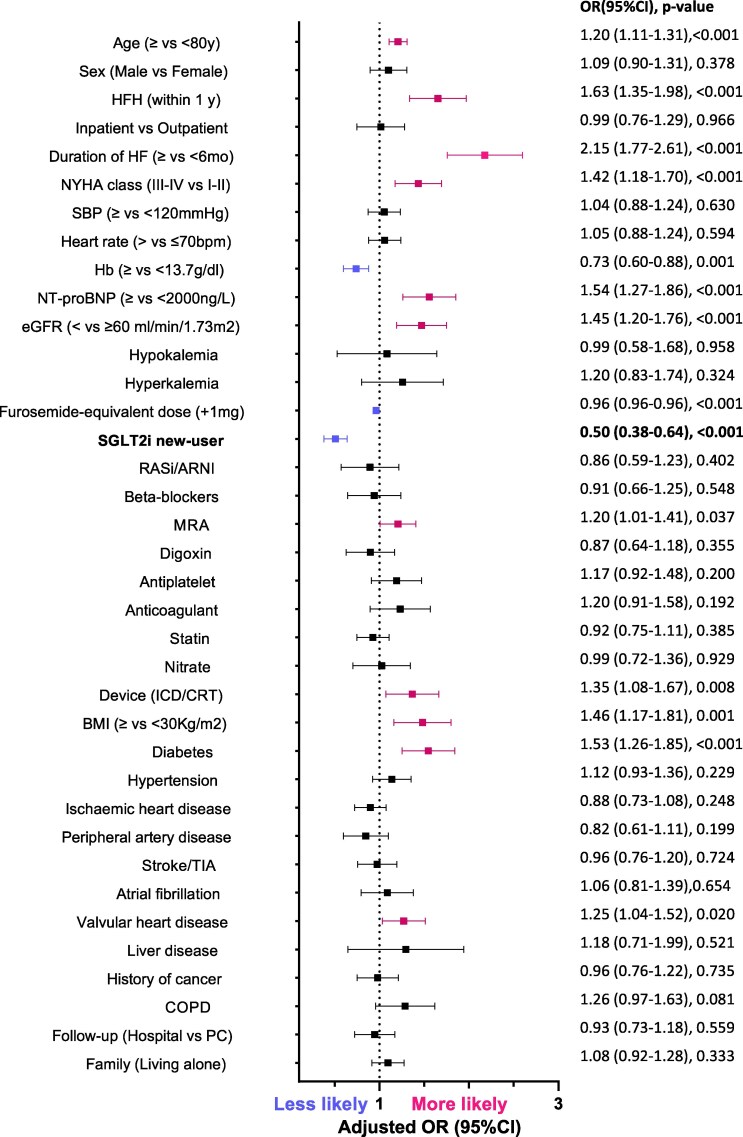
Predictors of increase in loop diuretic use/dosing at follow-up. ARNI, angiotensin receptor–neprilysin inhibitor; BMI, body mass index; CI, confidence interval; COPD, chronic obstructive pulmonary disease; CRT, cardiac resynchronization therapy; eGFR, estimated glomerular filtration rate; Hb, haemoglobin; HF, heart failure; HFH, heart failure hospitalization; ICD, implantable cardiac defibrillator; MRA, mineralocorticoid receptor antagonist; NYHA, New York Heart Association; NT-proBNP, N-terminal pro-B-type natriuretic peptide; OR, odds ratio; PC, primary care; RASi, renin–angiotensin system inhibitors; SBP, systolic blood pressure; SGLT2i, sodium–glucose co-transporter 2 inhibitors; TIA, transient ischaemic attack.

The multivariable logistic regression analysis showed consistent results after extensive adjustments (likelihood of loop diuretic discontinuation or dose decrease with SGLT2i initiation vs. no: adjusted OR 1.58, 95% CI 1.35–1.85, *P*-value<0.001, *Figure 3*; likelihood of loop diuretic initiation or dose increase with SGLT2i initiation vs. no: adjusted OR 0.50, 95% CI 0.38–0.64, *P* < 0.001, *Figure 4*). SGLT2i initiation was the strongest predictor of loop diuretic discontinuation/dose decrease and of no initiation/dose increase (*Figures 3* and *4*, respectively).

### Per-protocol analysis

Overall, 70 patients discontinued SGLT2i and 451 patients were initiated with SGLT2i during the 1-year follow-up, and therefore were excluded from the per-protocol analysis. The characteristics of patients discontinuing SGLT2i and detailed comparisons with those not discontinuing are reported in Supplementary material online, *Table S2*. No significant differences were observed.

At per-protocol analysis, both groups experienced a decrease in mean loop diuretic doses with a larger delta in those initiated with SGLT2i (see Supplementary material online, *Table S3*). The adjusted estimated mean difference was no longer significant in the per-protocol analysis (−2.2 [−5.1; 0.7] mg, *P*-value = 0.132) (see Supplementary material online, *Figure S3*).

The association between SGLT2i initiation and likelihood of decrease in loop diuretic use/doses was no longer significant in the multivariable model but the OR pointed out toward the same direction of association (adjusted OR 1.11, 95% CI 0.95–1.31, *P* = 0.197), whereas SGLT2i initiation was confirmed to be independently associated with less likelihood to increase in loop diuretic use/doses during follow-up (adjusted OR 0.44, 95% CI 0.34–0.57, *P*-value <0.001) (see Supplementary material online, *Tables S4 and S5*).

## Discussion

In the present study, we evaluated the association between SGLT2i initiation and changes in loop diuretics use over a 1-year follow-up in patients with HFrEF.

The main findings are the following (*Graphical abstract*):

1. Most patients were on loop diuretics at baseline, half of the patients maintained the same loop diuretic use and more patients reduced than increased use at the 1-year follow-up.
2. Among patients initiating vs. non-initiating an SGLT2i, discontinuation or dose reduction of loop diuretics was more likely, whereas a dose escalation or new initiation was less frequent. This was true in both unadjusted analysis and after adjusting for potential confounders that might affect diuretic need trajectory.

### Background loop diuretic therapy

We found that approximately three quarters of patients were receiving loop diuretics at baseline, with a mean furosemide-equivalent dose of 37.6 mg. The prevalence and dosing of loop diuretic therapy use in our cohort was consistent with what reported in other large cohorts of patients with HFrEF, predominantly with NYHA class II–III.^13,14^ Similarly, in DAPA-HF as well as in several recent trials, the median furosemide-equivalent dose was 40 mg.^8^ In the EMPEROR-Reduced trial, 13.2% of patients were not receiving loop diuretic therapy at baseline, while 20.0% were receiving <40 mg, 38.6% were receiving 40 mg, and 28.2% were receiving >40 mg of furosemide-equivalent daily dose.^7^ Of note, patients enrolled in real-world registries might be generally sicker than those enrolled in clinical trials. However, the application of the study specific inclusion criteria (e.g. the exclusion of patients with eGFR<20 mL/min/m^2^ and of those who died in the first 14-month follow-up) might have flattened the differences between real-world and clinical trials’ cohorts.

### Loop diuretic changes during follow-up

At follow-up, approximately half of the patients maintained the same loop diuretic dose. However, 39% patients experienced a dose reduction, and a minority (12%) had either a dose escalation or newly initiated loop diuretic therapy. In comparison, the Change the Management of Patients with Heart Failure Registry reported a loop diuretic dose increase in 23% of patients, with a median time to dose escalation of 5.3 months (IQR 1.9–11.1).^15^ In the larger ESC-HF Long-Term Registry, over ∼1-year of follow-up, loop diuretic doses were increased in 16% of patients, decreased in 8.3% and unchanged in 76%.^16^ The magnitude of our results with a high proportion of patients decreasing loop diuretic doses might be explained by the high proportion of guideline-directed medical therapy (GDMT) use in SwedeHF as compared to other Registries that finally improves the prognosis and reduces the diuretic need.^17,18^

### Loop diuretic changes in SGLT2i new users vs. controls

At multivariable linear regression analysis, initiation of SGLT2i was independently associated with a greater reduction in furosemide-equivalent dose at 1-year follow-up as compared with controls, with an adjusted estimated difference of −4 (95% CI −7 to −2 mg), equivalent to a 7% reduction of the baseline dose. This association was observed after adjustment for demographics, comorbidities, clinical and organizational characteristics, and concomitant treatment as well as furosemide-equivalent doses at baseline. Importantly, in our population the proportion of patients with good adherence (defined as ≥80% proportion of days covered) was high for SGLT2i (94%), as shown in a recent analysis.^19^ In addition, although the limited sample size warrants cautious interpretation, no evidence of effect modification by SGLT2i type was observed.

Alternative explanations for dose reduction, especially decrease in diuretic doses among patients included at the time of inpatient worsening HF and acutely increased in diuretic dose, cannot be excluded. However, the proportion of inpatients was consistent across the two groups. Thus, this phenomenon would be expected to affect both groups similarly and unlikely explains the observed differences between groups. Regression to the mean cannot be excluded. Baseline diuretic doses differed between groups, with higher values in the control group, which may have led to greater reductions independent of treatment.

As a comparison, in the EMPEROR-Reduced trial, empagliflozin was not superior to placebo in reducing time to loop diuretic de-escalation among patients on loop diuretics at baseline. However, it was associated with a higher likelihood of loop diuretic discontinuation among those receiving lower doses at baseline.^7^ A secondary analysis of DAPA-HF showed that the mean daily furosemide-equivalent dose did not differ between the dapagliflozin and placebo study arms after randomization, with most patients not experiencing a change in furosemide-equivalent dose at 2 weeks and at 2, 6, 12, and 18 months. Nevertheless, among the small proportion of patients changing loop diuretic doses, a decrease in dosing was more likely in the dapagliflozin as compared with the placebo arm (at 6 months, 10.4% vs. 7.3%, respectively, *P* < 0.001; at 12 months, 12.4% vs. 8.7%, *P* < 0.001).^8^ In addition, the authors showed that an increase in loop diuretic doses was less likely in the dapagliflozin group (at 6 months, 5.8% vs. 9.9%, *P* < 0.001; at 12 months, 10.2% vs. 14.2%, *P* < 0.001).^8^

In our analysis, initiation of SGLT2 inhibitors was independently associated with a significantly lower—approximately halved—risk of loop diuretic doses escalation or new initiation. These findings are consistent with results from the EMPEROR-Reduced trial, in which empagliflozin delayed the time to first dose escalation, among patients already receiving loop diuretics at baseline, and prolonged the time to loop diuretic initiation among those not on loop diuretics at baseline.^7^ In HF with mildly reduced or preserved EF, data from RCTs consistently showed that patients treated with loop or non-loop diuretic agents at baseline were less likely to require diuretic intensification, and more likely to experience diuretic discontinuation or dose reductions if randomized to SGLT2i.^9,10^ Between group differences in diuretic need was observed 60 days post-randomization.^20^ Our data add new evidence, showing a persistent lower need of loop diuretics at longer-term follow-up in patients with HFrEF. Also, SGLT2i initiation was one of the strongest predictors for reduction in loop diuretic need among the variables we considered. Importantly, we observed distinct patterns across baseline loop diuretic dose categories, with patients receiving low baseline doses of loop diuretics less likely increasing dose following SGLT2i initiation and those on intermediate doses showing the greatest reductions. In contrast, patients on high baseline doses (≥80 mg) exhibited smaller between-group differences that might be explained by more advanced HF or more advanced renal insufficiency.

The diuretic-sparing effect of SGLT2i is consistent with the benefits shown on major clinical endpoints. The initiation of an SGLT2i on top of other HF medications might lead to a reduction in required daily diuretic doses through several mechanisms, namely the demonstrated improvement in renal and cardiac function. HF hospitalizations usually lead to an increase in diuretic dosing at discharge, thus, the reduction of the risk of HF hospitalization by using SGLT2i might itself lead to a decrease in long-term diuretic doses. On the other hand, the direct effects of SGLT2i on diuresis and natriuresis are still not completely clear and cannot be excluded by our data.^4,21^ Nevertheless a careful revision of loop diuretic need at follow-up is advised.

### Implications for future clinical trials design

The findings of our study have important implications for the design of future RCTs. First, if diuretic requirement is included among eligibility criteria, the use of optimal GDMT and particularly SGLT2i may reduce the feasibility of patient enrolment by decreasing the need for loop diuretics. Second, it has been proposed that the intensification of oral diuretic therapy, that is surrogate of worsening HF, could be considered a relevant clinical endpoint, together with intravenous (i.v) diuretic administration.^22^ Indeed, previous studies showed that outpatient worsening HF events managed with oral diuretic intensification occur more frequently than those treated with i.v. therapy, and are similarly associated with increased morbidity and mortality.^16,22^ We found that initiating SGLT2i was independently associated with a lower risk of diuretic therapy escalation, indicating a reduction in the risk of outpatient worsening HF. We acknowledge that changes in diuretic doses might be driven by non-HF drivers, including changes in renal function, clinician practice, adherence, that are not considered in our analysis.

Similarly, in a secondary analysis of the DAPA-HF trial, dapagliflozin reduced the risk of worsening HF by 26%,^23^ a relative risk reduction comparable to that observed for HF hospitalization.^23^ Thus, our findings align with clinical trial results showing a reduced risk of in- and outpatient worsening HF with SGLT2i. The rate of events in our study supports the inclusion of oral diuretic intensification as an additional endpoint in order to increase event counts when designing RCTs.

## Limitations

Several limitations deserve acknowledgement. This was an observational study, and as such, although we performed extensive adjustments, it is prone to residual confounding. The exclusion of patients who died before 1-year follow-up assessment might have led to a selection bias (selection of healthier population). Misclassification of loop diuretics doses cannot be excluded: a patient could have a dispensed prescription without assuming the pill or assumed a lower dose. However, dispensation records (more than prescriptions) represent a pragmatic and widely accepted proxy for medication exposure in real-world studies. While escalation of oral loop diuretic therapy was used as a proxy for outpatient worsening HF, we acknowledge that dose adjustments may also be influenced by changes in renal function, changes in other GDMTs that were not assessed in our analysis. Also, the reasons behind loop diuretic dose changes were not assessed.

The recent introduction of SGLT2i among the foundational HFrEF therapies led to a significant proportion of patients initiating SGLT2i during the follow-up of interest. We performed a per-protocol analysis excluding cross-overs that overall confirmed the lower likelihood of loop diuretic use/doses increase after SGLT2i initiation.

Clinical and laboratory data including HF signs and symptoms and eGFR were not available at 1-year follow-up, preventing the possibility of reporting underlying causes of no change in loop diuretics doses. Also, the frequency of follow-up encounters was not reported and it could have influenced both the likelihood of SGLT2i initiation and subsequent loop diuretic titration, independent of HF trajectory. Finally, generalizability of our results is partially limited since patients enrolled in SwedeHF have different characteristics as compared with the overall HF population.^24^

## Conclusions

In a nationwide cohort of patients with HFrEF, SGLT2i initiation was associated with a lower need of loop diuretics and with a lower risk of outpatient worsening HF, defined as an escalation of oral loop diuretic therapy.

## Supplementary Material

pvag041_Supplementary_Data

## Data Availability

The data underlying this article will be shared upon reasonable request with the corresponding author.
