# Supplementary material for: Longitudinal loop diuretic use in patients with HFrEF initiated with sodium–glucose cotransporter 2 inhibitors
Source: Eur Heart J Cardiovasc Pharmacother. 2026 Jun 10;12(5):379–89. doi: 10.1093/ehjcvp/pvag041 (PMC13588644; doi:10.1093/ehjcvp/pvag041)
Supplement: Supplementary file 1 [file pvag041_supplementary_data.docx]

**Supplemental Material**

**Abbreviations**

ACEi, angiotensin-converting enzyme inhibitor; ARB, angiotensin receptor blocker; ARNi, angiotensin receptor–neprilysin inhibitor; BMI, body mass index; CI, confidence interval; COPD, chronic obstructive pulmonary disease; CRT, cardiac resynchronization therapy; EF, ejection fraction; eGFR, estimated glomerular filtration rate; Hb, haemoglobin; HF, heart failure; HFH, heart failure hospitalization; HFmrEF, heart failure with mildly reduced ejection fraction; HFpEF, heart failure with preserved ejection fraction; HFrEF, heart failure with reduced ejection fraction; ICD, implantable cardiac defibrillator; IQR, interquartile range; LVEF, left ventricular ejection fraction; LISA, Longitudinal integrated database for health insurance and labour market studies; MRA, mineralocorticoid receptor antagonist; NYHA, New York Heart Association; NPDR, National Prescribed Drug Registry; NPR, National Patient Register; NT-proBNP, N-terminal pro-B-type natriuretic peptide; obs, observation; OR, odds ratio; PAD, peripheral artery disease; SBP, systolic blood pressure; SD, standard deviation; SGLT2i, sodium-glucose co-transporter 2 inhibitors; T1DM, type 1 diabetes mellitus, VHD, valvular heart disease.

**Supplemental Table 1. Data sources and proportion of missing values.**

| **Variable** | **Data source** | **Missing (%)** |
| --- | --- | --- |
| **Demographics** |  |  |
| Sex | SwedeHF | 0 (0) |
| Age (years) | SwedeHF | 0 (0) |
| Calender year | SwedeHF | 0 (0) |
| Inpatient | SwedeHF | 0 (0) |
| HFH (within 12 months) | NPR | 0 (0) |
| Duration of HF (months) | NPR | 112 (2) |
| NYHA class | SwedeHF | 972 (15) |
| BMI (Kg/m2) | SwedeHF | 1122 (18) |
| SBP (mmHg) | SwedeHF | 240 (4) |
| Heart rate (beats/min) | SwedeHF | 271 (4) |
| eGFR (mL/min/1.73 m²) | SwedeHF | 2 (0) |
| Potassium | SwedeHF | 183 (3) |
| Haemoglobin (g/dl) | SwedeHF | 757 (12) |
| NT-proBNP (ng/L) | SwedeHF | 922 (14) |
| Loop diuretic | NPDR: ATC C03C ^a,b^ | 0 (0) |
| SGLT2i recommended for HFrEF (dapagliflozin, empagliflozin) | NPDR: A10BK01, A10BK03^a^ | 0 (0) |
| Other SGLT2i | NPDR: A10BK02, A10BK04, A10BK05, A10BK06, A10BK07, A10BK08, A10BK09 | 0 (0) |
| RASi/ARNi | SwedeHF | 6 (0) |
| Beta-blockers | SwedeHF | 4 (0) |
| MRA | SwedeHF | 5 (0) |
| Digoxin | SwedeHF | 7 (0) |
| Antiplatelet | SwedeHF | 6 (0) |
| Anticoagulant | SwedeHF | 7 (0) |
| Statin | SwedeHF | 8 (0) |
| Nitrate | SwedeHF | 7 (0) |
| CRT/ICD | SwedeHF | 5 (0) |
| Hypertension | SwedeHF | 140 (2) |
| Diabetes | SwedeHF | 64 (1) |
| Ischemic heart disease | Diagnosis in SwedeHF or in NPR: ICD-9/10 410-4, I20-5, Z951, Z955 Procedure FNA, FNB, FNC, FND, FNE, FNF, FNH, FNG ^c^ | 0 (0) |
| Atrial fibrillation/flutter | SwedeHF | 15 (0) |
| VHD | SwedeHF | 35 (1) |
| COPD | SwedeHF | 103 (2) |
| Liver disease | NPR: ICD-10 B18, I85, I864, I982, K70, K710, K711, K713-7, K72-4, K760, K762-9 ^c^ | 0 (0) |
| PAD | NPR: ICD-10 I70-3 ^c^ | 0 (0) |
| Previous stroke/TIA | NPR: ICD-9/10 430-4, 438, I60-4, I690-4 ^c^ | 0 (0) |
| History of cancer | NPR: ICD-10 C ^c^ | 0 (0) |
| Family type | LISA^d^ | 8 (0) |
| Follow-up location | SwedeHF | 2 (0) |
| Follow-up HF unit | SwedeHF | 198 (3) |

^a^ At baseline medications derived from dispensed prescriptions within +/- 2 months before/after index date

^b^ The dose taken by the patient was evaluated using the strength of the tablet prescribed multiplied by the number of pills the patients was prescribed to take each day. The latter is written in free text and an algorithm was derived to extract the dose prescribed. If there was no text or it was not possible to interpret the number of tablets/day, it was imputed using the mean number of pills taken per day for the posts with non-missing values with the same ATC code.

If multiple dispensations were registered during the 2 months preceding/following the baseline and follow-up assessment, the closest to the date of inclusion (for baseline use) and 1-year follow-up (for follow-up use) was chosen.

Limitations for dose calculation: the text extraction is sometimes a matter of interpretation. Further, in approximately 7% of the cases there is no text or no text indicating the number of pills that should be taken. For pills with combination substances the strength was not available. The % of combination pills was however low. The closest prescription to baseline and closest to 1 year were selected, however, the last prescription can be old and the doctor can verbally have instructed the patient to take a different dose/stop medication.

^c^ Derived from diagnoses/operations in all positions, in-patient and out-patient speciality care within 5 years prior to index (ICD-10)/1987 (ICD-9) unless stated otherwise (NPR includes data from hospitalization and out-patient speciality care but not primary care).

^d^ From the year prior to index date

**Supplemental Table 2. Comparison between patients who discontinued SGTL2i vs. those who did not discontinued SGLT2i.**

| **Variable** | **No discontinuation of SGLT2i** | **Discontinuation of SGLT2i** | **p-value** |
| --- | --- | --- | --- |
| **N** | **1195 (94.5%)** | **70 (5.5%)** |  |
| **Sex (male)** | 918 (76.8%) | 49 (70.0%) | 0.19 |
| **Age (years), median (IQR)** | 69.0 (61.0, 77.0) | 69.5 (61.0, 76.0) | 0.91 |
| **Calendar year** |  |  |  |
| **2020** | 217 (18.2%) | 18 (25.7%) | 0.11 |
| **2021** | 978 (81.8%) | 52 (74.3%) |  |
| **Inpatient** | 178 (14.9%) | 14 (20.0%) | 0.25 |
| **HF hospitalization (within 12 months)** | 589 (49.3%) | 28 (40.0%) | 0.13 |
| **Duration of HF (≥6 months)** | 479 (40.5%) | 32 (47.1%) | 0.28 |
| **NYHA class^a^** |  |  | 0.67 |
| **I-II** | 619 (60.4%) | 34 (57.6%) |  |
| **III-IV** | 406 (39.6%) | 25 (42.4%) |  |
| **BMI (≥ 30Kg/m2)^a^** | 322 (33.3%) | 13 (23.2%) | 0.12 |
| **SBP (mmHg), median (IQR)** | 120.0 (106.0, 132.0) | 120.0 (109.0, 132.0) | 0.59 |
| **Heart rate (bpm), median (IQR)** | 72.0 (63.0, 84.0) | 72.5 (64.0, 81.5) | 0.82 |
| **eGFR (ml/min/1.73m^2^), median (IQR)** | 74.1 (58.3, 90.3) | 78.5 (56.1, 95.3) | 0.48 |
| **eGFR (<60ml/min/1.73m^2^)** | 317 (26.9%) | 19 (27.5%) | 0.91 |
| **Potassium categories** |  |  | 0.19 |
| **Normokalemia** | 1093 (93.0%) | 67 (98.5%) |  |
| **Hypokalemia** | 38 (3.2%) | 0 (0.0%) |  |
| **Hyperkalemia** | 44 (3.7%) | 1 (1.5%) |  |
| **Haemoglobin (≥13.7g/dL)^a^** | 600 (55.9%) | 28 (43.1%) | 0.043 |
| **NT-proBNP (≥2000ng/L)^a^** | 468 (44.0%) | 29 (44.6%) | 0.93 |
| **RASi/ARNI^a^** | 1162 (97.2%) | 67 (95.7%) | 0.26 |
| **Beta-blockers^a^** | 1140 (95.4%) | 66 (94.3%) | 0.67 |
| **MRA^a^** | 833 (69.7%) | 45 (64.3%) | 0.56 |
| **Digoxin^a^** | 97 (8.1%) | 2 (2.9%) | 0.11 |
| **Antiplatelet^a^** | 384 (32.1%) | 22 (31.4%) | 0.90 |
| **Anticoagulant^a^** | 623 (52.1%) | 38 (54.3%) | 0.73 |
| **Statin^a^** | 699 (58.5%) | 35 (50.0%) | 0.16 |
| **Nitrate^a^** | 52 (4.4%) | 2 (2.9%) | 0.55 |
| **ICD/CRT** | 205 (17.2%) | 12 (17.1%) | 1.00 |
| **Hypertension^a^** | 765 (64.0%) | 41 (58.6%) | 0.36 |
| **Diabetes^a^** | 445 (37.2%) | 26 (37.1%) | 0.99 |
| **Ischaemic heart disease^a^** | 584 (48.9%) | 34 (48.6%) | 0.96 |
| **Atrial fibrillation^a^** | 566 (47.4%) | 33 (47.1%) | 0.97 |
| **Valvular heart disease^a^** | 244 (20.4%) | 19 (27.1%) | 0.18 |
| **COPD^a^** | 97 (8.1%) | 13 (18.6%) | 0.003 |
| **Liver disease^a^** | 30 (2.5%) | 4 (5.7%) | 0.11 |
| **PAD** | 88 (7.4%) | 6 (8.6%) | 0.71 |
| **Previous stroke/TIA** | 146 (12.2%) | 7 (10.0%) | 0.58 |
| **History of cancer** | 111 (9.3%) | 4 (5.7%) | 0.31 |
| **Family type** |  |  | 0.47 |
| **Cohabiting** | 666 (55.8%) | 36 (51.4%) |  |
| **Living alone** | 527 (44.2%) | 34 (48.6%) |  |
| **Follow-up location** |  |  | 0.89 |
| **Primary care/other** | 54 (4.6%) | 3 (4.3%) |  |
| **Hospital** | 1111 (95.4%) | 67 (95.7%) |  |

**Supplemental Table 3. Diuretic dose changes from baseline to follow-up stratified by SGLT2 initiation after the exclusion of patients who discontinued SGLT2i (n=70) or initiated SGLT2i (n=451) during follow-up (per-protocol analysis).**

| **Variable** |  | **No SGLT2i** | **SGLT2i new-users** | **Between group difference** | **p-value** |
| --- | --- | --- | --- | --- | --- |
| N |  | 5172 (81.2%) | 1195 (18.8%) |  |  |
| On loop diuretic at baseline |  | 3945 (76.3%) | 787 (65.9%) | -10.4% (-13.3%; -7.5%) | <0.001 |
| Furosemide-equivalent dose baseline (mg), mean (SD) |  | 37.6 (28.0) | 32.1 (28.8) | -5.5 (-7.3; -3.8) | <0.001 |
| On loop diuretic at follow-up |  | 2517 (48.7%) | 388 (32.5%) | -16.2% (-19.2%; -13.2%) | <0.001 |
| Furosemide-equivalent dose follow-up (mg), mean (SD) |  | 24.7 (29.7) | 16.5 (27.7) | -8.2 ( -10.0; -6.3) | <0.001 |
| Difference dose (follow-up - baseline) (mg), mean (SD) |  | -13.0 (33.2) | -15.6 (30.2) | -2.7 ( -4.7; -0.6) | 0.011 |
| Difference dose in percentage (%), mean (SD) |  | -30 (60) | -40 (60) | -4.2 (-7.8; -0.6) | 0.024 |
| Difference dose (follow-up - baseline), categories |  |  |  |  | <0.001 |
|  | No changes | 2339 (45.2%) | 603 (50.5%) | 5.2% (2.1%; 8.4%) |  |
|  | Reduced/withdrawn | 2114 (40.9%) | 500 (41.8%) | 1.0% ( -2.1%; 4.0%) |  |
|  | Increased/new initiation | 719 (13.9%) | 92 (7.7%) | -6.2% (-8.0%;-4.4%) |  |

**Supplemental Table 4. Per-protocol regression analysis for decrease in diuretic doses after the exclusion of patients who discontinued SGLT2i (n=70) or initiated SGLT2i (n=451) during follow-up (per-protocol analysis).**

| **Variable** | **Unadjusted OR (95%CI)** | **p-value** | **Adjusted OR (95%CI)** | **p-value** |
| --- | --- | --- | --- | --- |
| **SGLT2i new-user** | 1.04 (0.92-1.18) | 0.540 | 1.11 (0.95-1.31) | 0.197 |

**Supplemental Table 5. Per-protocol regression analysis for increase in diuretic doses after the exclusion of patients who discontinued SGLT2i (n=70) or initiated SGLT2i (n=451) during follow-up (per-protocol analysis).**

| **Variable** | **Unadjusted OR (95%CI)** | **p-value** | **Adjusted OR (95%CI)** | **p-value** |
| --- | --- | --- | --- | --- |
| **SGLT2i new-user** | 0.52 (0.41-0.65) | <0.001 | 0.44 (0.34-0.57) | <0.001 |

**Supplemental Figure 1. Flowchart of the study population.**

*ATC codes for other SGTL2i: A10BK02 canagliflozin; A10BK04 ertugliflozin; A10BK05 ipragliflozin; A10BK06 sotagliflozin; A10BK07 luseogliflozin; A10BK08 bexagliflozin; A10BK09 enavogliflozin.

**Supplemental Figure 2. Time window definition.**

Prescriptions for SGLT2 inhibitors and diuretics, obtained from the National Prescribed Drug Registry (NPDR), were evaluated at baseline and at 12-month follow-up using a 4-month time window. For each time point, the prescription closest to month 0 and month 12 was selected.

**Supplemental Figure 3. Changes in diuretic doses from baseline to 12 months, stratified by SGLT2i initiation (linear regression analysis) after the exclusion of patients who discontinued SGLT2i (n=70) or initiated SGLT2i (n=451) during follow-up (per-protocol analysis).**

Furosemide-equivalent doses

Furosemide-equivalent doses (mg)

|  | No SGLT2i | SGLT2i |
| --- | --- | --- |
| Baseline | 37.6 | 32.1 |
| Follow-up | 24.7 | 16.5 |
